# Inclusive education: A transformation and human rights agenda under spotlight in South Africa

**DOI:** 10.4102/ajod.v4i1.183

**Published:** 2015-11-04

**Authors:** Mbulaheni Maguvhe

**Affiliations:** 1 Department of Inclusive Education, School of Educational Studies, University of South Africa, South Africa

## Abstract

This study investigated the progress made in the implementation of inclusive education as a transformation and human rights tool since its inception in 2001. The study was conducted upon realising that most people underestimate the transformation and human rights value that inclusive education strives to maintain. The total number of participants interviewed was 84. Data was collected using semi-structured interview schedules for the teachers and community members, whereafter it was presented in thematic sections and qualitatively examined for meaning. The results showed that participants comprising teachers and community members do not know or understand the transformational and human rights value of inclusive education. The participants seemed to be equally aware of inclusive education, but they rated its success and value differently. The participants concurred that the philosophy of inclusive education was noble, but they differed regarding the extent to which it had transformed, added value or played an advocacy role in the lives of learners and the community at large over the years.

## Introduction

The practice of inclusive education in South Africa is underpinned by six democratic assertions: all children and youth can learn under conducive learning circumstances and need unwavering, ongoing support; there ought to be relevant support structures, ideal systems and methodologies that enable such support in the education system; learners are different and the differences must be both acknowledged and respected; learning does not only take place in the formal school, but also at home and in the community; changes have to be made to attitudes, behaviour, teaching methods, curriculum and environment to meet the diverse and sometimes complex learning needs of all learners; and all such efforts ought to be aimed at minimising barriers to learning while maximising the participation of all learners in the curriculum and culture of their educational institutions (Department of Education [DoE] 2001:6). McConkey (2003:10) argues that inclusive education promotes ‘full participation and equality’ through enabling children with disabilities from restrictive family backgrounds a chance to interact with others and participate in the life of their communities. Thus, inclusive education is a transformation tool, a human right and a democratic way of understanding values and forming beliefs, which welcome (and actually celebrate) human diversity (Rieser 2005; Swart & Pettipher 2005:8).

The democratic affirmations that justify inclusive education also draw the parameters of the operational meaning of the philosophy, thereby defining the upper limits of the possibility of its implementation in the education system. The meaning of inclusive education that has been embedded in the minds of many is that it is about including the traditionally excluded, such as learners with sensory, learning and physical disabilities. It does not explicitly include learners who face barriers that are due to poverty and low socio-economic status - yet, they should be considered (Prinsloo 2005:28). This view has narrowed the national focus on moving the inclusive education agenda. The democratic angle of inclusive education has been applied to learners with learning, sensory and physical disabilities. For schools to be truly inclusive, they need to consider whether all those learners who have never been part of the mainstream have been welcomed, and seek to make the environment truly welcoming to all, irrespective of their significant differences. Through true inclusive participation, children experiencing barriers to learning realise genuine chances for information sharing and develop self-representation skills (Maguvhe 2015:133).

A more detailed understanding must include learners in particularly precarious life circumstances and adverse socio-economic conditions so that all the learners feel welcome, irrespective of their ethnic groups, languages, religions, social classes, gender, sexuality or disabilities (Swart & Phasha 2005:214). Thus, questions on power dynamics, diversity and difference are attended to when there is contentment among learners, and the focus is no longer on adapting the environment for only certain groups.

The machinery that should move inclusive education forward has to be guided by the National Department of Education’ s (2002) notion of the implementation entailments:

Inclusive education is about the whole education system from the national, provincial and district offices of the Department of Education, to individual schools and their communities, and to individual teachers and learners. (p. 4)

Teachers must make sure that the philosophical orientation to inclusive education and its practice reaches every department of education and every learner. Teachers are important catalysts for inclusive education – there are some who have questioned the success of the inclusive education drive since its inception in 2001, mainly because the teachers are not adequately trained (Chataika *et al.* 2012; Dalton, Mckenzie & Kahonde 2012), or they are not confident that they have mastered the content of their training in inclusive education (Walton *et al.* 2014). To achieve efficacy and confidence, teachers need ongoing training (Wiazowski 2012). The focus is on the measures that could bring about effective inclusive education in view of the fact that inclusive schooling is still teething in the 14th year since its implementation. The inertia that is being experienced in the widespread implementation of inclusive education is reason enough for anyone to suspect that the current methods of educating people to develop a conviction in inclusive education are not capable of changing people towards voluntary participation in the process.

Notwithstanding the definition people use to include all who should learn in a democratic school environment, the application of the inclusive education policy depends on the positive actions of all role players. The perspectives of those involved in the inclusive education initiative have to be transformed before they could become advocates of the agenda for inclusivity. A change in perspective, through transformative learning, comprises three processes an individual should undergo psychologically:

1. the learners must undergo shifts in understanding themselves as a result of new information
2. the learners must experience a shift in their personal beliefs (convictions)
3. the learners must change their behaviours to adopt a matching lifestyle (ed. Mezirow 2000).

These three processes are under the learners’ realm of control since it is the learners who decide on personal actions.

Transformative learning applies mostly to adults, as they can engage their old beliefs using new information and resolve to believe differently from a certain kind of ‘knowing’ that was a part of them (ed. Mezirow 2000). This implies the need for a strong conviction with regard to the importance of inclusive education on the part of policymakers and teachers.

The complex issue about the drive for inclusive education is that there are vast methodological and implementation differences across countries. In some countries, the placement process for learners with special learning needs is clearer than in other countries. In the United States of America (USA), the process of placing children with special learning needs in schools of choice is clearly outlined in sequential steps for parents to follow. Most children with special learning needs are educated in mainstream schools. Parents have the responsibility to follow the guidelines provided in making a choice of an appropriate school, bearing in mind the culture that they want their child to experience, as well as the capacity of the school to accommodate the learner's unique learning needs (United States Department of Education 2007).

In the United Kingdom (UK), mainstream schools absorb the majority of students with disabilities. These schools absorb about 64% of blind and partially sighted students. There are also resourced schools and special schools. Schools that are privately owned mostly provide residential facilities, while those funded by local authorities are day schools. Only academies are funded by the Department for Education (DfE). The choice of a school is the responsibility of parents, but they are guided by a local authority list of suitable schools (Royal National Institute for the Blind 2015). The Royal National Institute for the Blind emphasises the need for parents to visit every school listed by the local authority to ensure that the school is able to satisfy the learning needs of the child.

The placement process in Kenya is different from practices in the USA and the UK since student identification, assessment and placement are the responsibility of Educational Assessment and Resource Centres (EARCs), a division within the Kenyan Ministry of Education (CBM 2015). The role of parents in the placement process is not clearly defined in the Kenyan student placement process.

In South Africa (SA), as in the USA and in the UK, it is every child's democratic right to attend a school that is nearest to his or her home. The difference is that in SA there are few full-service schools that can accommodate learners with special learning needs, giving mainstream schools the latitude to refuse admission of learners with special learning needs (if they feel they are not well-equipped to provide for the child's unique learning needs). Besides, parents are not provided with clear step-by-step guidelines on how to choose appropriate mainstream schools for their children. Parents are also not adequately educated on the need to play an active role in the selection of appropriate schools for their children.

In practice in SA, therefore, the right to education does not extend to the right to make a choice of a specific school in the child's home area. This means that, although pupils have the right to attend any of the schools in their residential area, nothing could be done if these schools refuse to admit them. Van Rooyen, Le Grange and Newmark (2002) observe that South African education policies are contradictory to each other such that they do not align well enough to enable smooth implementation of inclusive education. This makes more poignant the fact that the struggle for education as a human rights agenda may not be over yet. Pather (2011) recommends constant revisits to the policy of inclusion to fine-tune mechanisms for implementation.

## Research objectives

The major objective of the study was to investigate the extent to which inclusive education has transformed the South African school system to take stock of the progress made in implementing inclusive education as a transformation and human rights tool.

## Theoretical framework

The present study is underpinned by the systems theory, which is a science of organisations and their wholeness (Higgs & Smith 2006:27). Human beings, like the schools that teach them, are systems. The development of individuals to their optimum level of complexity is closely related to *what* they learn and *how* they learn in their institutions of learning. The development of schools into comprehensive and optimally beneficial entities is of paramount importance. Systems influence each other, so that people have an influence on the development of schools, religions, industry and hospitals as much as those institutions influence people. It is important for people to advance the utility value of schools as regards their inclusivity so that the schools would help to transform human society positively.

### Aim of the study

The aim of the study was to understand the views of school-level stakeholders on the progress made in the implementation of inclusive education.

### Research questions

Below are the research questions the researcher asked the participants:

1. To what extent, has the education system transformed in respect of inclusivity since 1994?
2. Is the South African education system now better equipped to accommodate learners with diverse learning needs than it was before democratic rule?
3. How do role players in education contribute to the national transformation agenda?
4. How can the transformation and human rights agenda be enhanced on inclusion of learners with disabilities?

## Methodology

### Design

The study used a qualitative survey research design. The design was preferred for its suitability to the collection of opinions about a phenomenon (Gray 2004; McMillan & Schumacher 2010).

### Sample

The sample comprised 60 teachers (28 males and 32 females), 12 members of school governing bodies (six males and six females) and 12 representatives of community-based disabled persons’ organisations (seven males and five females). The particular teachers included in the sample were granted permission to participate in the study by their principals, and they were selected because they were recommended by peers as having good background knowledge on inclusive education. The sample of teachers was drawn from six mainstream schools from four provinces, namely Limpopo, Gauteng, North West Province and the Free State. Those schools were purposely sampled as they are designated ‘full-service schools’, which should be offering inclusive education by now. Randomly picking any mainstream schools would not have shown the progress of implementing the inclusive education master plan as articulated in Education White Paper 6 of 2001. The sample of community members (SGB and DPO representatives) was drawn from each school community such that there were two members of the school governing body (SGB) and two representatives of disabled persons’ organisations (DPOs) from each school community. Each SGB and DPO nominated his or her preferred representatives. The total number of participants interviewed was 84.

### Instruments

Two sets of semi-structured interview schedules were used to collect data: one set was for gathering data from teachers and the other for gathering data from members of SGBs and DPOs. The trustworthiness of the instruments was tested through a pilot study run among teachers, SGB representatives and representatives of community-based DPOs from four schools, which were not used for the final data collection process (Golafshani 2003).

### Data collection procedures

The study was informed by the views expressed by 60 teachers from six mainstream schools and 24 community members, comprising 12 SGB members and 12 representatives of local DPOs. The researcher used 12 focus groups - two per school. For each school, one group was composed of eight teachers (to capture the views of teachers) and the other focus group of six participants comprised two teachers, two SGB members and two representatives of a DPO (to capture mainly the views of community members). In this way, it was possible to cater for the literacy levels and experiences of the teachers and community members using two different instruments in terms of how they would understand the questions and formulate their responses – most notably on responses made by community members.

### Data analysis

Data were presented in thematic sections that arose from responses and interviews. The data were then qualitatively examined for meaning. The responses of teachers and community members were interpreted with reference to documented national policies and published international developments in inclusive education, in light of grounded theory and social ecological models (Swart & Pettipher 2005:6) applied to education.

### Ethical issues

All teachers and community members participated voluntarily. All participants were free not to participate and to withdraw at any time without reprisals, and they did not have to answer questions that made them feel uncomfortable. The participants were also assured of the confidentiality of the inputs and their anonymity in the whole research process. It was made known to the participants that data were collected only for the purposes of the present study as described.

## Discussion

The discussion below follows the four research questions on inclusive education and the transformation and human rights agenda, presented earlier.

### Inclusive education and the transformation and human rights agenda

Question 1: To what extent has the education system transformed in respect of inclusivity since 1994?

Over three-quarters (59) of the participants were of the view that the transformation and human rights agenda in SA was not on track. They ascribed this to the fact that there were only a few designated full-service schools in the Department of Education's White Paper 6. Only these few designated full-service schools have the capacity to provide for the needs of learners facing various barriers to learning, which is to the detriment of the majority of learners. The following verbal quotes support these findings:

‘Transformation towards inclusive education … no! We need to admit all learners. How can our school admit and cater for them while we are not trained, equipped and better prepared, and without any facelift of the infrastructure?’ (Teacher from school A)

‘We are aware that … learners are members of our communities and [*by*] admitting them for the sake of admitting, we shall be committing a serious crime. I think if we do not teach them like any child we … admit, we have failed’. (Teacher from school A)

‘It is not on track, no. I myself, I only hear on [*the*] radio – especially [*on*] the educator/learner support programme - that it is on track. Mind you, these are departmental officials. They cannot say it is not on track’. (Community member from school A)

‘We have learners with disabilities in our community. Eish, I bat, the receive low quality education [*sic*]. Teachers are not au fait with methodologies of including them. They fail. As a community, we are to blame as well. We do not ask for ideas from other communities where this system … works well’. (Community member from school C)

Van Louw and Beets (2008) maintains that striving for academic excellence in higher education is not restricted to South Africa, ‘but is in actual fact a global phenomenon that is primarily manifested in the manner in which knowledge production is achieved’. This supports the notion that even inclusive education schools ought to transform. Through systems theory it is evident that incompletely developed schools have a retrogressive influence on human society. As human society has pointed to the benefits of inclusivity, schools must be developed to a point where they advance the social realities of people as systems that are in need of optimum development (Higgs & Smith 2006:30). Problems causing the slow pace at which inclusive education is being implemented in SA need to be carefully analysed so that solutions can be found.

### Accommodating learners with diverse learning needs

Question 2: Is the South African education system now better equipped to accommodate learners with diverse learning needs than it was before democratic rule?

Participants indicated that schools in urban areas were somewhat better equipped to accommodate learners with diverse learning needs than those in rural areas. This statement is supported by the Department of Education (2011) when it argues that one of the most significant barriers to learning remains the inability of learners to access the educational provision that does exist, as well as their inability to access other services that contribute to the learning process. The previous cited author went further, arguing that, in most instances, the inability to access educational provision results from inadequate or non-existent services and facilities, which are key to participation in the learning process. Participants’ observations concur with Mukuria and Obiakor’ s (2004) observation that resources for programme implementation were often scarce in poorer communities in the African diaspora. Hence, rural schools had fewer resources than urban schools. It is important for stakeholders to examine alternative solutions to the problem and equip rural schools, so that they offer optimum benefit to their learners.

According to participants, access to services and facilities in urban areas could be attributed to the availability of things such as accessible transport, buildings that meet universal access standards, educated parents who are involved in the education of their children, learners’ access to information due to facilities like tape aids for the visually impaired and print-disabled, and braille services that can convert material into accessible formats. However, participants indicated that mainstream schools did not utilise these resources to accommodate learners with diverse needs. The following verbal quotes illustrate the findings:

‘The situation in urban areas is far … better when compared to the rural one. There, support is in abundance. Learners are ferried to and from schools by public transport’. (Teacher from school A)

‘Educated parents would fight fiercely for their children's rights if they [*were*] trampled upon. However, in rural areas, we see the opposite because the illiteracy level among parents is still high’. (Teacher from school B)

‘Most of the things that make education accessible in rural areas are not there. Can you imagine, some learners still use donkey carts or ox wagons as a means of transport?’ (Teacher from school C)

‘Learners do not have access to specialised machines and books for their studies and do not even know that there are facilities in big cities where they can send them for conversion’. (Teacher from school D)

The community members in the study revealed that inclusive education had not changed the profile of the rural child as much as that of the urban child. The following verbal quotes illustrate these findings:

‘It will take *mengwagangwaga*, I mean years if not decades for the rural schools to be better equipped to accommodate all learners. How will schools transform if as parents we do not add our voices to … advocating for the provision of resources’. (One community participant from school A)

‘*Aralizeileludzi zwi hone, ndi zwithukhuthukhunahone azwi vhonalinaluthihi* [If resource provision in regards resources is there, it is very minimal and is not noticeable]’.(One community participant from school B)

‘*Ritshisedzambalombalo, vhanavha re zwikolonizwannyinannyi, vhatshevhanevhalavhaneravhadivha* [When we look at statistics, the number of learners at mainstream schools is still the same we know]’. (One community participant from school C)

‘*Tshandukoyavhukuma I tutuwedzwana u di sendekakhazwikonazwileludzizwapfunzozwoteaho* [True change is influenced and hinges on relevant resources and the right infrastructure]’. (One community participant from school D)

‘A diversity of learners and changing the face of schools? Just a wild dream! Rural areas will never be on par with urban areas when it comes to the admission of learners. Our schools do not have resources suited for their intricate needs’. (One community participant from school D)

‘Teachers are in the dark in [*regard to*] the implementation of inclusive education. Actually, the majority of them see it as a fearful monster coming to devour them. If rural areas can receive the kind and level of support that urban areas receive, I believe we would see or notice a significant change in the learner profile’. (One community participant from school B)

### Contribution by role players to the national transformation agenda

Question 3: How do role players in education contribute to the national transformation agenda?

According to the Ministry of Education Singapore (2015), there are many stakeholders in education, each of whom needs to play their role effectively and efficiently in order to help all children learn better and reach their full potential. For example, parents must support schools in their efforts to educate the child, teachers must care deeply for the character and moral development of students through both words and actions, communities must welcome and encourage the youth to be involved in the community activities, and so forth.

There were 74 participants that were of the view that stakeholders were not playing their part in the transformation agenda. Apparently, stakeholders were passing the buck thinking it was somebody else's responsibility to play a meaningful role in the education of learners with disabilities in mainstream schools. The following verbal quotes illustrate these findings:

‘It is a pity, [*the*] National Department thinks that the Province is the right structure and stakeholder to see that inclusion materialises. The Province also thinks [*it is*] the District, and the District Office thinks it is the Circuit… It is a circus’. (Teacher from school B)

‘Schools expect too much from their communities just because in the African culture, any adult member of society is a parent and should play that parental role of teacher/guide’. (Teacher from school C)

‘Things are easier said than done. When you listen to authorities, it is all systems go pertaining to inclusive education. Everybody is playing his part. However, the reality is that we are just scratching the surface when it comes to stakeholder participation’. (Teacher from school C)

‘I suspect this could be due to unclear roles each and every stakeholder has to play or [the] thinking [*that*] somebody else [*will*] play dual or triple roles. For instance, as a teacher, my role is to teach and not make provisions for resources, policy, etc.’ (Teacher from school A)

‘Our roles and power differ remarkably. For transformation to take place in earnest, it is incumbent upon us all to do what is expected of us or [*what we are*] mandated to do’. (Teacher from school D)

In line with systems theory, it is the efforts of key stakeholders, such as the Department of Basic Education, to examine the resourcing woes of full service schools openly and to provide the inputs required by the education system so that it would be able to make a meaningful output in the form of genuine inclusive education, which in turn benefits the South African society (Higgs & Smith 2006:30)

### The enhancement of the transformation and human rights agenda

Question 4: How can the transformation and human rights agenda be enhanced to include learners with disabilities?

There were 67 participants who were in agreement that factors such as clear inclusive education guidelines and policies, adherence to implementation timeframes, high conviction in the policy among key reformers, and active participation of communities in inclusive education could enhance it tremendously. The following verbal quotes illustrate the findings:

‘Well-crafted policies with tight timelines and good monitoring and evaluation instruments could lead to transformation being realised’. (Teacher from school A)

‘If implementers of policy understand that which they have to implement, I cannot foresee any hindrance for transformation to take off the ground’. (Teacher from school C)

‘This should be coupled with zeal, devotion, dedication, commitment, and of course, proper control of events and situations’. (Teacher from school C)

According to Education Transformation (2015), all systems and schools have assets that could be developed and enhanced to achieve significant improvements in students’ learning and school performance. In this context, best practices need to be harnessed, teaching methodology must be enriched, parents should be engaged in new ways, and appropriate technology should be available to provide access and improve quality of learning for more students.

## Results

The study revealed that transformation towards inclusive education is slow. The participants identified a lack of training as one of the reasons for the reluctance to participate in the programme. The result concurs with Mezirow's (ed. 2000) view that adults only adopt change wholeheartedly if they have successfully undergone transformative learning. Transformative learning ensures change in personal beliefs around any philosophy, and with the new convictions comes behaviour change, which features changes, such as a new (matching) lifestyle. Following this theory, it will be meaningless to discuss inclusive education and the democratic observation of all learners without putting transformative learning in place at workshops and awareness raising courses, which are meant for practitioners at national, provincial and district education offices. With their newly found conviction for successful learning, they could develop school-level staff on the entailments of voluntary participation in daily practices, which will ensure successful inclusive education. The other setback is a lack of resources, which Maguvhe (2005) also alluded to. Genuine practice is the embodiment of inclusivity as a human right.

The study further revealed that changes in the outlook of learners in terms of their diversity in a school, their schooling environment, and the contents of their school bags were more noticeable in urban areas than in rural areas. One of the findings of the study revealed that the positive impact of educational provisioning is more felt in urban than in the rural areas. The Department of Education (2001:6) specifies that support structures will be established for inclusive education, which will attend to adequate material provisioning and proper practitioner training for the necessary change to take place in attitudes, behaviour, teaching methods, curricular and the environment. There is a slow movement towards the inclusivity agenda, which means that some diverse and sometimes complex learning needs of different learners are not yet adequately met. Systems theory informs the need for open examination of the school system in order to identify implementation blind spots and to correct them quickly, as well as for the social benefits that come with school improvements on inclusivity (Higgs & Smith 2006:30).

The study further revealed that the key stakeholders did not perform as expected, as if they were not sure of their roles. This apparent uneasiness with designated roles could have been the reason for them expecting too much from one another. The role of everyone in the inclusive education playing field is important, according to the National Department of Education’ s (2002:4) observation. It is, therefore, expected that everyone's role should be crystal clear to avoid role conflict. Even the learner's role must be clarified through good, evidence-based teaching practices. The literature points out that teachers are not adequately trained (Chataika *et al.* 2012; Dalton *et al.* 2012) to meet the challenges of inclusive education. It is more informative to investigate whether higher authorities at district, provincial and national education offices are adequately trained to articulate downstream what policy dictates.

Lastly, the study revealed that the policy on inclusive education was not well-aligned with its implementation tools, making it difficult to enable teachers to train and communities to see hope in a changing society. A lack of touch with the skills development context for transformative education is evident among teachers (Walton *et al.* 2014). Some teachers have become defiant or uncooperative regarding their work as agents of inclusive education. They would need transformative training to elevate inclusive education to the level of a human right.

## Recommendations

Inclusive education seems to be going nowhere. It is, therefore, recommended that:

1. A massive awareness campaign is driven in rural areas so that communities will know and understand their role in supporting learners with disabilities.
2. Support structures must be reviewed so that even the community is officially mandated to play a meaningful role, with its responsibilities clearly identified in the education policy so that the community can be held accountable if it is not playing the desired role.
3. Teachers and school management teams must be trained and encouraged to enrol with higher education institutions to acquire qualifications that are relevant to inclusive education, apart from the crash courses they attended. Such comprehensive training and ongoing professional peer support and collaboration are critical components of transformational learning.
4. All matters must be considered when planning inclusive education - especially those discussed under theme 4 - for inclusion to work.
5. The ‘*bathopele*’ principle is applied at inclusive education schools. This will attract learners in rural areas and make them understand that they will receive quality education regardless of where they are.

## Conclusion

Stakeholders need to ensure that there is co-operation, teacher/learner support systems, community involvement and a proper system of inclusive education, which is implemented by means of the teacher and learner population with the involvement of non-governmental and non-profit organisations, institutions of higher learning, research institutions, early childhood development centres, community education training centres and so forth. All of us need to participate and play our part in inclusive education.

In order for teachers and learners to form new beliefs around inclusive education and to effect real change for learners to ‘live’ inclusive education, there is a need for significant changes in the way that people in educational administration at national, provincial, district and school-level understand themselves in relation to inclusive education.
